# Early life stress and body-mass-index modulate brain connectivity in alcohol use disorder

**DOI:** 10.1038/s41398-024-02756-8

**Published:** 2024-01-20

**Authors:** Khushbu Agarwal, Paule V. Joseph, Rui Zhang, Melanie L. Schwandt, Vijay A. Ramchandani, Nancy Diazgranados, David Goldman, Reza Momenan

**Affiliations:** 1 Section of Sensory Science and Metabolism, National Institute on Alcohol Abuse and Alcoholism, National Institutes of Health, Department of Health and Human Services, Bethesda, MD 20892 USA; 2 National Institute of Nursing Research, National Institutes of Health, Department of Health and Human Services, Bethesda, MD 20892 USA; 3 Laboratory of Neuroimaging, National Institute on Alcohol Abuse and Alcoholism, National Institutes of Health, Department of Health and Human Services, Bethesda, MD 20892 USA; 4 Office of the Clinical Director, National Institute on Alcohol Abuse and Alcoholism, Bethesda, MD 20892 USA; 5 Human Psychopharmacology Laboratory, National Institute on Alcohol Abuse and Alcoholism, Bethesda, MD 20892 USA; 6 Laboratory of Neurogenetics, National Institute on Alcohol Abuse and Alcoholism, Rockville, MD 20892 USA; 7 Clinical NeuroImaging Research Core, National Institute on Alcohol Abuse and Alcoholism, Bethesda, MD 20892 USA

**Keywords:** Addiction, Molecular neuroscience

## Abstract

Early life stress (ELS) significantly increases susceptibility to alcohol use disorder (AUD) by affecting the interplay between the executive and the salience networks (SNs). The link between AUD and higher body-mass index (BMI) is known, but we lack understanding of how BMI impacts the relationship between ELS and brain connectivity in individuals with AUD. To bridge this gap, we investigated the main and interaction effects of ELS and BMI on brain connectivity in individuals with AUD compared to non-AUD participants (*n* = 77 sex-matched individuals per group). All participants underwent resting-state functional magnetic resonance imaging, revealing intriguing positive functional connectivity between SN seeds and brain regions involved in somatosensory processing, motor coordination and executive control. Examining the relationship of brain connectivity with ELS and BMI, we observed positive associations with the correlations of SN seeds, right anterior insula (RAIns) and supramarginal gyrus (SMG) with clusters in motor [occipital cortex, supplementary motor cortex]; anterior cingulate cortex (ACC) with clusters in frontal, or executive, control regions (middle frontal gyrus; MFG, precentral gyrus) that reportedly are involved in processing of emotionally salient stimuli (all |β | > 0.001, |*p* | < 0.05). Interestingly, a negative association of the interaction effect of ELS events and BMI measures with the functional connectivity of SN seeds ACC with decision-making (MFG, precentral gyrus), RAIns and RSMG with visuo-motor control regions (occipital cortex and supplementary motor cortex) (all |β | = −0.001, |*p* | < 0.05). These findings emphasize the moderating effect of BMI on ELS-associated SN seed brain connectivity in AUD. Understanding the neural mechanisms linking BMI, ELS and AUD can guide targeted interventions for this population.

## Introduction

Early life stress (ELS), as determined by self-reported traumatic childhood events, such as emotional abuse, severe family conflict, domestic violence and bullying, has been linked to increased vulnerability to the development of alcohol use disorder (AUD) [1–3]. Individuals with AUD and obesity exhibit poor decision-making abilities, which has been linked to disruptions in the salience network (SN) assessed in resting state functional MRI studies [4, 5]. This maladaptive decision-making has been attributed to difficulties in switching between executive and salience networks in alcohol drinkers [6]. Furthermore, a decrease in resting state functional connectivity within the default mode network (DMN) and an increase in connectivity between the reward, limbic and SNs have been reported in obesity [7]. Accordingly, studies have extensively examined the relationship between heavy alcohol consumption and increased body weight over the past years [8].

It has been speculated that heavy alcohol consumption can lead to a higher body-mass index (BMI) via insulin resistance [9, 10]. Although the evidence for a comorbidity between obesity and AUD is conflicting [11], the clinical relationship between these two disorders is complex and both are affected by common aspects of vulnerability to adverse events, as well as subsequent events whereby excess alcohol consumption can lead to both weight gain and to weight loss [8, 12, 13]. Previous studies have shown that ELS is associated with alterations in brain structure and function, including reduced *centrality* (defined as the impact of a particular region of the brain on the transmission and exchange of information within extensive networks of the brain) in SN regions, such as the anterior insula (AIns) and dorsal anterior cingulate cortex (ACC) [14–16].

Despite extensive research conducted on the topic, there is still insufficient evidence of any association of ELS-related events and BMI on alterations of brain connectivity of salience, executive, somatosensory and impulse control networks in individuals with AUD. In our earlier study using data from the human connectome project (HCP) we found notable associations between chronic alcohol consumption, BMI and the decision-making capabilities for monetary rewards in people with high-risk AUD and obesity symptoms [17]. Expanding on these findings, our present aim is to investigate the similarities in the association between BMI and ELS and the resting-state seed-based functional connectivity of decision-making related regions in individuals diagnosed with AUD compared to those without an AUD diagnosis. We hypothesize that in individuals with AUD compared to those without AUD, early life stress and BMI may exhibit a positive association with the connectivity of the salience network node to brain regions involved in executive control and decision-making processes.

## Materials and methods

### Participants

The study cohort, as presented in Table 1, was comprised of 154 sex-matched participants categorized into those with (*n* = 77) and without (*n* = 77) a diagnosis of AUD. All participants underwent resting state functional MRI scans. AUD participants in this study were treatment-seekers and were inpatients admitted in the NIAAA clinic for treatment and their resting state functional MRI scans were obtained following detoxification and withdrawal period from alcohol. Diagnosis of AUD was made via the Structured Clinical Interview for the Diagnostic Statistical Manual (DSM)-IV or DSM-5 (SCID) [18–20]. For the current analysis, individuals who were diagnosed with alcohol dependence or abuse via SCID-IV were considered to have an AUD. Daily alcohol consumption in the 90 days preceding the study was assessed using the timeline follow-back (TLFB) method [21]. Individuals with substance use disorders other than AUD and nicotine dependence were excluded. Patients were allowed to smoke during their stay but were requested not to smoke or remove their nicotine patch two hours prior to their MRI scan. The study was approved by the Institutional Review Board of the National Institutes of Health, and all participants provided written informed consent to participate.

**Table 1:** Demographic and clinical characteristics of participants (*n* = 154).

	AUD cohort (*n* = 77)	Non-AUD cohort (*n* = 77)	Test Statistics *T* test or, Mann–Whitney for continuous/Chi-square for categorical variables
Age (years) (mean ± SD)	47.9 ± 11.3	45.4 ± 11.2	p = 0.03
Sex			
Males *n* (%)	39 (49.4%)	39 (49.4%)	χ2 = 0; df = 1; *p* = 1.00
Females *n* (%)	38 (50.6%)	38 (50.6%)	
Years of Education	13.5 ± 2.7	16.5 ± 3.7	*p* < 0.001
Household Income (Median)	30K-39, 999	50K-74, 999	χ2 = 22.5; df = 8; *p* = 0.002
Total AUDIT Score	29.2 ± 5.9	2.8 ± 2.2	*p* = 0.00
Smoking status			
Yes, *n* (%)	50 (64.9%)	1 (1.3%)	χ2 = 70.4; df = 1; *p* < 0.001
No, *n* (%)	27 (35.2%)	76 (98.7%)	
Ethnicity, *n* (%)			
Non-Hispanic or Latino	70 (90.9%)	68 (88.3%)	χ2 = 3.17; df = 2; *p* = 0.21
Hispanic or Latino	5 (6.5%)	9 (11.7%)	
Unknown/not reported	2 (2.6%)	0 (0.0%)	
*Race, n (%)*			
White	38 (49.4%)	30 (39.0%)	χ2 = 11.07; df = 5; *p* = 0.05
Black/African American	24 (31.2%)	31 (40.3%)	
Asian	1 (1.3%)	9 (11.7%)	
American Indian or Alaska Native	1 (1.3%)	0 (0.0%)	
Multiracial	8 (10.4%)	4 (5.2%)	
Unknown Race	5 (6.5%)	3 (3.9%)	
Medications During Inpatient Stay	Antihypertensive agent, angiotensin-converting-enzyme (ACE) inhibitor; alpha or, beta-adrenergic agonist; angiotensin receptor blocker; anti-manic agent, anticonvulsant agent; benzodiazepine agent; antidepressant agents (e.g., Selective Serotonin Reuptake Inhibitor (SSRI), Tricyclic Antidepressant (TCA) Agent); smoking cessation agent; antiemetic agent, serotonin receptor antagonist 5-HT3; attention deficit hyperactivity disorder therapy agent, norepinephrine reuptake inhibitor; calcium channel blocker agent; diabetes mellitus therapy agent; gastric acid secretion inhibitor, proton pump inhibitor; etc.	NA	-
BMI (Kg/m^2^)	26.5 ± 5.7	26.1 ± 4.9	*p* = 0.86
ELS events (Median; IQR)	3.6; 5.8	1.0; 2.9	*p* < 0.001

NA: not available; Household Income: <$10,000 = 1, $10K-$19,999 = 2, $20K-$29,999 = 3, $30K-$39,999 = 4, $40 K $49,999 = 5, $50K-$74,999 = 6, $75K-$99,999 = 7, >=$100 000 = 8; length of AUD history (difference in age of AUD onset and current age). Here, * represents statistical significance with *p* value < 0.05.

### Body-mass index (BMI)

BMI for all participants in the study was calculated by dividing their body weight by the square of their height (Kg/m^2^). Measurements of both body weight and height were recorded when participants enrolled in the NIAAA natural history study. The continuous BMI variable was utilized to conduct further analysis to identify the relationship between BMI, and ELS on brain connectivity patterns in AUD vs. non-AUD participants.

### Early life stress (ELS) events

ELS was operationalized using self-report questionnaire which consists of 19 standard life event items experienced in their early life as a child (up to age 18 years). The participants’ responses were recorded as yes/no for each life event including emotional, sexual, and physical abuse, as well as violence, negligence, parental divorce, surgery, parental death, separation and so forth [22, 23]. The sum of responses for all domains was used to create a complete ELS_events score (with a maximum score of 19; *Mean* = 3.58, *SD* = 3.2). Emotional abuse, severe family conflict, domestic violence and bullying were the most reported events.

### Resting-state functional MRI (rsfMRI) data acquisition and preprocessing

Resting-state fMRI (rs-fMRI) scans were acquired from patients during the inpatient treatment phase when they were stabilized and not experiencing stress or acute withdrawal symptoms. Patients’ withdrawal scores were assessed using the Clinical Institute Withdrawal Assessment for Alcohol-revised (CIWA-Ar), a 10-item scale [24]. To be eligible for rs-fMRI scans, patients had to have an average CIWA-Ar score below 8, which typically occurs at 1 week after admission, and the scans were done during weeks 2 or 3 of their inpatient stay. The scans were conducted at the NIH NMR Center, utilizing a Siemens 3 T MRI Skyra scanner with a 20-channel head coil. Participants were instructed to keep their eyes open and remain alert during the ten-minute period of rs-fMRI data collection, with no additional stimuli presented. The rs-fMRI scans were acquired utilizing an echoplanar-imaging pulse sequence (TR: 2000 ms, TE: 30 ms, FA: 90°, FOV: 240 × 240 mm, 3.8 mm slice thickness, multi-slice mode: interleaved). A high-resolution T1-weighted MPRAGE (TR: 1900 ms, TE: 3.09 ms, FA: 10°, FOV: 240 × 240 mm, 1 mm slice thickness) was obtained for registration purposes. Preprocessing of the data was carried out using the CONN toolbox (version 18.b; https://www.nitrc.org/executedashboard/?group_id=279), a Matlab-based toolbox for functional connectivity analysis (http://www.nitrc.org/projects/conn) [25], including realignment and unwarp, slice-timing correction, outlier identification, and normalization. Artifact detection was performed based on scan-to-scan differences in the global signal (z-value threshold 5) and subject motion parameters (threshold 0.9 mm) using the ‘art’ software for artifact rejection (www.nitrc.org/projects/artifact_detect/), with identified outlier scans included as first-level covariates.

### Functional connectivity

To analyze rs-fMRI data, we used the CONN toolbox (18.b) with full width at half maximum spatial smoothing of 8 mm. To minimize effects of head motion, we regressed out principal components associated with segmented white matter and cerebrospinal fluid using CompCor [26], as well as twelve motion regressors (3 rotational, 3 translational, and their derivatives) calculated from CONN image preprocessing. The data were filtered using a band-pass filter of 0.008-0.09 Hz to eliminate very-low-frequency drift and high frequency noise, and linear trends were removed. We used a continuous squashing function (*i*.*e*., despiking) to further minimize the influence of potential outlier scans. Global BOLD signal was not regressed out to avoid the mathematical introduction of negative correlations [27].

We conducted a seed to voxel (whole brain) resting state connectivity analysis to investigate the influence of ELS on the connectivity of SN seed regions and the rest of the brain. The seeds were selected a priori based on our hypothesis of strong interaction of SN with executive function networks in addictive conditions [28–30]. The seeds were defined based on the anatomical FSL Harvard-Oxford atlas, which is the default atlas utilized for segmentation during the CONN processing procedure. We included the anterior insula, anterior cingulate cortex, and inferior parietal cortex (supramarginal gyrus) as seed regions associated with the SN. A separate model was created for the left and right structures for each seed. We extracted the mean time series of the seed region from their preprocessed functional data and calculated Pearson’s correlation coefficients for the connection between the seed and voxel for each participant. To enable further analyses, we transformed the resulting values into normally distributed Z-scores using the Fisher transformation. The identified correlations are presented in the results section.

### Statistical analyses

**Fig. 1:**
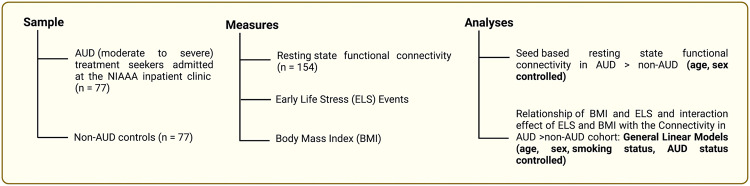
The study flow listing the sample, measures and analytic techniques. The alcohol use disorder (AUD) and non-AUD population. The measures included early life stress (ELS) events, body-mass-index (BMI) and resting-state functional connectivity (rs-fMRI). The main analyses consisted of seed-based rs-fMRI to identify connectivity patterns in individuals with AUD, as well as General Linear Models to identify relationships of connectivity differences with ELS events and BMI as well as the interaction effects of ELS events and BMI measures.

To compare the demographic and clinical characteristics of our study groups (AUD vs. non-AUD), we utilized Student’s *t*-tests and Mann–Whitney tests for continuous variables, and chi-squared tests for categorical variables. The age and sex-controlled connectivity coefficients between the SN seeds and significant clusters in the AUD compared to non-AUD group were extracted from CONN. We considered connection-level False Discovery Rate (FDR)-corrected *P* values < 0.05 as significant [31]. We then employed general linear model (GLM) to investigate the relationship of ELS events and BMI and the interaction effect of ELS events and BMI measures with the correlations of seed and significant clusters (interpreted as connectivity, the dependent variable) and included ELS events, BMI, age, sex, smoking status, and AUD status as fixed factors. For all descriptives and regression models, we used SPSS 22 (IBM Corp., Armonk, NY). Figure 1 provides an overview of the study flow, including participant measures and statistical approach used to investigate the study hypothesis.

## Results

### Characteristics of the sample: demographic and clinical

There was a significant difference in age distribution between the AUD and non-AUD cohorts (test statistic = −5.6; *p* < 0.001). The full BMI and ELS distribution of the AUD and non-AUD cohorts are reported in Supplementary Fig. S1. The data show a significant difference in distribution of ELS across the AUD vs. non-AUD cohorts (test statistic = −5.5; *p* < 0.001), while BMI distribution was not significantly different across the study cohorts (test statistic = 0.18; *p* = 0.86).

Furthermore, the percentage of smokers was significantly higher in the AUD cohort (64.9%) compared to the non-AUD cohort (1.3%; *p* < 0.001). Individuals with AUD had a lower level of education (mean years of education, 13.5 ± 2.7) and had low household income (30K-39, 999) compared to participants without AUD [(mean years of education, 16.5 ± 3.7; *p* < 0.001) (household income, 50K-74, 999; *p* = 0.002)]. No significant difference in race and ethnicity was identified between the AUD and non-AUD groups (refer to Table 1).

### Resting state fMRI

#### Seed-based voxel connectivity (SBVC) in AUD compared to non-AUD

**Fig. 2:**
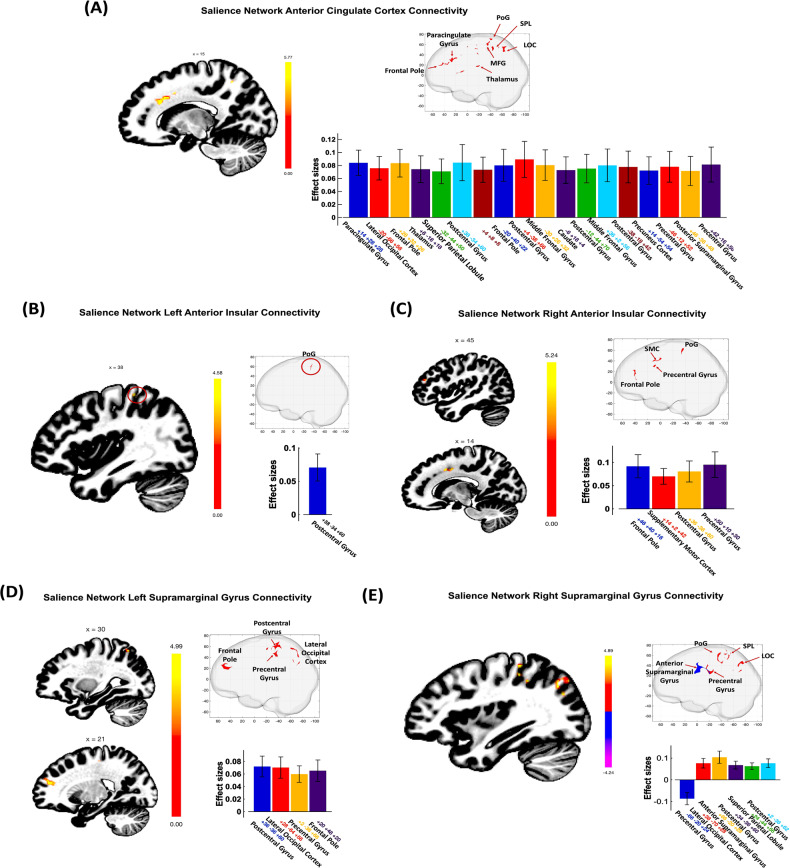
Functional connectivity pattern in AUD compared to non-AUD. **A**–**D** Significant Seed-to-Voxel connections representing salience network seed regions (anterior cingulate cortex, anterior insula left/right and supramarginal gyrus left/right). Labels provided for perspective reference are as follows: SMC Supplementary Motor Cortex, MFG Middle Frontal Gyrus, PoG Postcentral Gyrus, LOC Lateral Occipital Cortex, SPL Superior Parietal Lobule; cluster of significant activation at the peak-wise *P*_FWE_ < 0.001/cluster size *P* < 0.05 FDR corrected level. Directions of connectivity are noted in Table 2.

Several clusters displayed altered connectivity with our seed regions, including the left/right SMG, left/right AIns, and ACC, following adjustment for age and sex in the AUD compared to the non-AUD cohort. For further information on the connected clusters, please refer to Table 2. Additionally, a visual representation of the extent of significance can be found in Fig. 2.

**Table 2:** Associations of ELS events and BMI with seed-to-voxel functional connectivity in AUD compared to Non-AUD controls.

Seed		Connectivity cluster						Adjusted Estimate (β), (95% CI), *p* value	Adjusted Estimate (β), (95% CI), *p* value	Adjusted Estimate (β), (95% CI), *p* value
Label	Lat.	Label	Lat.	x	y	z	k	Relationship of ELS events with Connectivity in AUD>non-AUD cohort	Relationship of BMI with Connectivity in AUD>non-AUD cohort	Interaction ELS events*BMI
Salience Network_ACC		Paracingulate Gyrus	R	+14	+28	+28	66	0.01, (−0.01, 0.02), 0.41	0.001, (−0.002, 0.004), 0.54	0.00, (−0.001, 0.00), 0.48
		Lateral Occipital Cortex	L	−20	−66	+56	65	0.01, (−0.01, 0.03), 0.24	0.001, (−0.002, 0.004), 0.54	0.00, (−0.001, 0.00), 0.27
		Frontal Pole	R	+28	+32	+26	58	0.01, (−0.02, 0.03), 0.63	0.002, (−0.002, 0.01), 0.33	0.00, (−0.001, 0.001), 0.60
		Frontal Pole	L	−20	+40	+22	35	0.003, (−0.02, 0.03), 0.84	0.002, (−0.002, 0.01), 0.25	0.00, (−0.001, 0.001), 0.63
		Thalamus	R	+06	−18	+18	57	0.01 (−0.01, 0.03), 0.35	0.002, (−0.002, 0.01), 0.34	0.00, (−0.001, 0.01), 0.27
		Superior Parietal Lobule	L	−32	−44	+50	54	0.01, (−0.01, 0.03), 0.15	0.003, (−0.01, 0.03), 0.08	0.00, (−0.001, 0.00), 0.23
		Postcentral Gyrus	R	+38	−34	+60	38	0.02, (−0.003, 0.05), 0.07	0.01, (0.00, 0.01), 0.03*	−0.001, (−0.002, 0.00), 0.12
		Postcentral Gyrus	L	−12	−44	+70	28	0.01, (−0.01, 0.04), 0.27	0.003, (−0.001, 0.01), 0.13	0.00, (−0.001, 0.00), 0.23
		Middle Frontal Gyrus	R	+36	+02	+56	26	0.02, (−0.01, 0.03), 0.23	0.01, (0.003, 0.01), <0.001*	0.00, (−0.001, 0.00), 0.27
		Middle Frontal Gyrus	L	−30	+26	+32	32	0.03, (0.01, 0.01), 0.01*	0.01, (0.001, 0.01), 0.01*	−0.001, (−0.002, −7.67E–5), 0.03*
		Caudate	L	−06	+18	+04	30	0.01, (−0.01, 0.03), 0.17	0.001, (−0.003, 0.004), 0.69	0.00, (−0.001, 0.00), 0.20
		Precuneus Cortex	-	+14	−54	+54	23	0.01, (−0.01, 0.03), 0.12	0.001, (−0.002, 0.01), 0.51	−0.001, (−0.001, 0.00), 0.10
		Precentral Gyrus	L	−46	−12	+52	23	0.02, (0.002, 0.04), 0.03*	0.004, (−2.3E–5, 0.006), 0.04*	−0.001, (−0.001, 6.09E–5), 0.04*
		Precentral Gyrus	R	+42	−16	+58	21	0.03, (0.004, 0.05), 0.02*	0.01, (0.003, 0.01), 0.002*	−0.001, (−0.002, 0.00), 0.02*
		Posterior Supramarginal Gyrus	R	+46	−36	+48	22	0.00, (−0.02, 0.02), 0.96	0.001, (−0.003, 0.01), 0.65	−7.6E-05, (−0.001, 0.001), 0.85
Salience Network_AIns	L	Postcentral gyrus	R	+38	−34	+60	39	−0.01, (−0.02, 0.01), 0.49	−0.001, (−0.004, 0.002), 0.61	0.00, (0.00, 0.001), 0.46
	R	Frontal Pole	R	+46	+40	+16	61	0.01, (−0.01, 0.03), 0.40	0.004, (−0.001, 0.01), 0.08	0.00, (−0.001, 0.001), 0.38
		Supplementary Motor Cortex	R	+14	+02	+42	54	0.02, (0.01, 0.03), 0.006*	0.001, (−0.002, 0.006), 0.03*	−0.001, (−0.001, 0.00), 0.01*
		Postcentral Gyrus	R	+36	−36	+60	52	0.01 (−0.01, 0.03), 0.28	0.001, (−0.002, 0.01), 0.44	0.00 (−0.001, 0.00), 0.31
		Precentral Gyrus	R	+50	+10	+30	34	0.02, (−0.002, 0.05), 0.06	0.002, (−0.003, 0.01), 0.47	−0.001, (−0.002, 5.37E-5), 0.06
Salience Network_SMG	L	Postcentral Gyrus	R	+36	−36	+60	195	0.01 (−0.01, 0.02), 0.52	9.9E-5, (−0.003, 0.003), 0.94	0.00 (−0.001, 0.00), 0.46
		Lateral Occipital Cortex	R	+28	−64	+56	133	0.01, (−0.01, 0.02), 0.39	0.001, (−0.002, 0.004), 0.57	0.00, (−0.001, 0.00), 0.49
		Precentral Gyrus	R	+02	−38	+66	121	−0.01, (−0.02, 0.01), 0.29	−0.001, (−0.003, 0.001), 0.32	0.00, (0.00, 0.001), 0.28
		Frontal Pole	R	+20	+48	+20	106	−0.001, (−0.01, 0.01), 0.87	−0.002, (−0.01, 0.001), 0.18	−4.29E-5, (−0.001, 0.001), 0.89
	R	Precentral Gyrus	L	−66	−20	+24	149	−0.01 (−0.01, −0.04), 0.17	0.00, (−0.004, 0.01), 0.88	−0.001, (−0.002, 0.00), 0.08
		Lateral Occipital Cortex	R	+38	−70	+28	120	0.02, (0.003, 0.04), 0.02*	0.01, (0.001, 0.01), 0.009*	−0.001, (−0.002, −2.75E–5), 0.04*
		Anterior Supramarginal Gyrus	R	+66	−20	+28	96	−0.002, (−0.03, 0.02), 0.87	0.002, (−0.002, 0.01), 0.33	3.07E-5, (−0.001, 0.001), 0.95
		Postcentral Gyrus	R	+34	−36	+60	81	0.008, (−0.01, 0.02), 0.37	0.00, (−0.003, 0.003), 0.81	0.00, (−0.001, 0.00), 0.51
		Superior Parietal Lobule	R	+24	−44	+56	80	0.00, (−0.01, 0.01), 0.69	−0.001, (−0.003, 0.002), 0.67	0.00, (−0.001, 0.00), 0.60
		Postcentral Gyrus	R	+02	−36	+62	76	0.001, (−0.02, 0.02), 0.92	0.001, (−0.002, 0.01), 0.39	−8.67E–5, (−0.001, 0.001), 0.82

Statistics and descriptive for seed-to-voxel connectivity analyses. All clusters were false discovery rate (FDR) corrected at *p* < 0.05 and peak value family wise error (FEW) corrected.Estimates reported for ELS events and BMI relationship were after adjustment for age, sex, smoking status, and AUD status. Here *indicates statistical significance with *p* < 0.05.

*k* number of voxels in the cluster, *Lat. (R/L)* laterality of brain region (Right/Left), *SD* standard deviation, *SMG* Supramarginal Gyrus, *ACC* Anterior Cingulate Cortex, *AIns* Anterior Insula, *AUD* Alcohol Use Disorder, *BMI* Body-Mass Index.

The ACC seed region demonstrated positive connectivity with clusters in both the right and left frontal pole, postcentral gyrus, precentral gyrus, and middle frontal gyrus (MFG), as well as the left lateral occipital cortex (LOC), superior parietal lobule (SPL), and caudate. Additionally, it exhibited connections with the right thalamus, posterior supramarginal gyrus, and precuneus cortex. The left AIns seed exhibited positive connectivity with a cluster in the left postcentral gyrus. Conversely, the right AIns seed displayed positive connections with the right frontal pole, supplementary motor cortex, precentral gyrus, and postcentral gyrus (see Table 2; Fig. 2A–C).

The left SMG seed exhibited positive connectivity with various clusters, including the right LOC, postcentral gyrus, precentral gyrus, and frontal pole. In contrast, the right SMG seed demonstrated negative connectivity with the left precentral gyrus, while displaying positive connectivity with the right LOC, anterior supramarginal gyrus, postcentral gyrus, and SPL (refer to Table 2; Fig. 2D, E).

#### Association of ELS events and BMI with SBVC in individuals with AUD versus non-AUD

Following adjustment for age, sex, smoking status, and AUD status in our GLM analysis, we identified several associations of SBVC in the AUD versus non-AUD cohort with both ELS events and BMI. Specifically, we observed positive associations between increased occurrences of ELS events and BMI measure with the connectivity of the ACC with left MFG [ELS events: (β = 0.03; CI = 0.01, 0.01; *p* = 0.01), BMI: (β = 0.01; CI = 0.001, 0.01; *p* = 0.01)] and bilateral precentral gyrus [ELS: left, (β = 0.02; CI = 0.002, 0.04; *p* = 0.03), right, (β = 0.03; CI = 0.004, 0.05; *p* = 0.02); BMI: left, (β = 0.004; CI = 2.3E–5, 0.006; *p* = 0.04), right precentral gyrus (β = 0.01; CI = 0.003, 0.01; *p* = 0.002)] clusters. Functional connectivity of RAIns seed with right supplementary motor cortex cluster was also significantly positively correlated with both ELS events (β = 0.02; CI = 0.01, 0.03; *p* = 0.006) and BMI measures (β = 0.001; CI = 0.002, 0.01; *p* = 0.03). Functional connectivity of RSMG seed with right LOC cluster was also significantly positively correlated with both ELS events (β = 0.02; CI = 0.003, 0.04; *p* = 0.02) and BMI measures (β = 0.01; CI = 0.001, 0.01; *p* = 0.009).

We also identified an interaction effect of ELS events and BMI measures on these connectivity patterns in AUD vs. non-AUD cohort. ELS events*BMI was negatively associated with the connectivity patterns: ACC seed connection with left MFG (β = −0.001; CI = −0.002, 7.67E–5; *p* = 0.03) and bilateral precentral gyrus [left, (β = −0.001; CI = (−0.001, 6.09E–5; *p* = 0.04); right, (β = −0.001; CI = 0.002, 0.00; *p* = 0.02)]); RAIns seed connection with right supplementary motor cortex (β = −0.001; CI = 0.001, 0.00; p = 0.01); RSMG seed connection with right LOC (β = −0.001; CI = 0.002, −2.75E–5; *p* = 0.04) (see Table 2 for details).

## Discussion

The current study sought to investigate the functional connectivity of SN seed in AUD versus non-AUD participants and their association with history of ELS events and BMI measures. As predicted, SBVC in AUD versus non-AUD study participants revealed several positive connectivity patterns of SN seeds, including ACC, bilateral AIns and bilateral SMG with whole brain clusters in somatosensory and motor coordination areas (such as the bilateral LOC, supplementary motor cortex, postcentral gyrus, and supramarginal gyrus); frontal, or executive control regions (e.g., key nodes of the fronto-parietal network: MFG, precentral gyrus, SPL); and nodes in posterior DMN (precuneus, thalamus and caudate).

The connectivity patterns identified in participants with AUD (*vs*. non-AUD) in our study is in alignment with a previous report in participants with AUD wherein increased within and between SN, DMN and executive control networks functional connectivity were noted in AUD compared to healthy controls using a whole-brain probabilistic independent component analysis approach [32]. This heightened functional connectivity between the SN seed regions, specifically the insula and anterior cingulate cortex (ACC) and the visual cortex (LOC) and middle frontal gyrus (MFG), was also detected in a study by Han et al. at a moderate alcohol dose [33]. The observed increased connectivity between the SN seed regions and somatosensory and motor coordination areas may be attributed to an enhanced involvement of the SN in detecting and assigning emotional significance to relevant sensory stimuli. This interpretation finds further support in reports of increased connectivity between the ACC and the sensorimotor network [34] and is consistent with the visuomotor effects associated with alcohol [35, 36]. In line with previous reports suggesting a crucial role of DMN nodes, such as precuneus in social and self-related cognitive processes [37, 38], the increased ACC-precuneus coupling identified in our study might pertain to heightened self-awareness and emotional response to negative social stimuli in AUD subjects. This, in turn, could potentially increase impulsive decision-making and drinking behaviors as a way of regulating these emotions in individuals with AUD (vs. non-AUD). Additionally, as previously highlighted in our own studies [32, 39], the heightened functional connectivity identified in individuals with AUD compared to those without AUD may indicate a potential neural mechanism of compensation or adaptation following long-term alcohol exposure, wherein the structural damage resulting from chronic alcohol use [40–42] is potentially restored through the dynamic coupling of related networks, including the SN, motor coordination networks and DMN.

In our population which comprised of moderate-to-severe AUD and non-AUD subjects, BMI distribution did not reveal a significant difference; however, a clear difference in the distribution of ELS events between patients with AUD and without AUD was seen. This hints that the relationship of BMI and ELS in the context of AUD may not be straightforward, and there may be other factors that have a stronger impact on the association between ELS and BMI in individuals with AUD. Consequently, upon examining the association of history of ELS events and BMI with the identified connectivity in AUD (vs. non-AUD) subjects, we identified several clusters that were associated with both ELS and BMI increases.

Notably both ELS events and BMI were positively associated with the functional connectivity between SN seeds, RAIns and RSMG with clusters in motor [occipital cortex, supplementary motor cortex], ACC with clusters in frontal, or executive, control regions (MFG, precentral gyrus) that reportedly are involved in processing of emotionally salient stimuli [43–46]. Exposure to stress during critical periods of brain development has been demonstrated to modify connectivity patterns and heighten the risk of developing AUD. Likewise, numerous studies provide evidence that experiencing ELS has harmful effects on individuals and enhances their susceptibility to alcohol use in adulthood [2, 47–51]. Moreover, exposure to a series of ELS events leads to modifications in connectivity of brain regions associated with emotion, self-regulation and cognition, including nodes within the fronto-limbic networks, such as the mPFC, ACC, amygdala and orbitofrontal cortex [52–54]. Children between the ages of 9 and 16 who were exposed to various stress events, such as conventional crimes, child maltreatment, peer/sibling victimization and sexual victimization, were found to have a reduced functional connection between their SMG and PCC [55]. Moreover, it is noteworthy that exposure to acute stress has been linked with elevated functional connectivity between the nodes of the default mode and SNs in healthy adults and adolescents [55, 56]. Considering the diverse range of ELS events encountered by the participants in our study, spanning from a single event to as many as nineteen stressors, it is conceivable that the directional connectivity between the SN and various brain regions was influenced by the cumulative extent of their early life stress experiences. Moreover, these connectivity patterns exhibited a positive association with the elevation of BMI, suggesting heightened functional connectivity between the SN and brain areas implicated in decision-making processes and the coordination of visual and motor functions in individuals with AUD. The findings suggest two potential scenarios: either ELS contributes to overeating in individuals with AUD, or ELS-induced overeating could heighten their vulnerability to excessive alcohol consumption. Consequently, the connections of the SN with regions governing decision-making (fronto-parietal) and the coordination of visual and motor functions (lateral occipital cortex and supplementary motor cortex) might encounter further impact in individuals with AUD due to the co-occurring effects of early life stress and heightened BMI, thereby leading to irregular processing of salient stimuli. To test this hypothesis, we assessed the interaction effect of assessment of the interaction effect between the frequency of ELS events and BMI measurements with the identified patterns of functional connectivity in individuals with AUD as compared to those without AUD.

Interestingly, we identified a negative association of the interaction effect of ELS events and BMI measures with the functional connectivity of SN seeds ACC with decision-making (MFG, precentral gyrus), RAIns and RSMG with visuo-motor control regions (LOC and supplementary motor cortex). Both pre-clinical and clinical studies have identified the contribution of ELS in increasing the risk for obesity [57–59] and AUD [48, 60, 61], which was attributed to persistent overactivation of the hypothalamic-pituitary-adrenal (HPA) axis [62], dysregulation of the mesolimbic dopamine functions [63, 64] and an imbalance in connectivity patterns of salience, emotion and somatosensory networks [65]. Nonetheless, none of these studies demonstrated the combined relationship of increased occurrence of ELS events and BMI measures on brain connectivity in adults with AUD compared to those without AUD. Our results suggest that the increase in both ELS events and BMI disrupts the connectivity of SN with decision-making and visuo-motor coordination regions, potentially amplifying impulsive decision-making and compromising self-control behaviors. These altered behaviors, influenced by ELS and exacerbated by an increase in BMI, may be interpreted as the underlying drivers for the worsening of early life stress history related AUD [66].

The present study offers intriguing insights into the intricate relationship between ELS, BMI and AUD and the connections of salience network seeds with the whole brain. These findings corroborate our initial hypothesis, which postulates that heightened BMI might influence the connectivity patterns between the SN and specific brain regions responsible for regulating executive control and impulsive behaviors, particularly in individuals diagnosed with AUD and a history of early life stress. Moreover, the notable detrimental effect on the connectivity of SN seeds with fronto-parietal and visuo-motor coordination networks, specifically in AUD individuals (vs. non-AUD), because of increased BMI stemming from a higher frequency of stressful events during early life, implies a potential neurobiological mechanism through which the combination of elevated BMI and a history of early life stress contributes to alterations in the functional connectivity of crucial brain networks in these individuals. Further exploration of these intricate associations can significantly enhance our understanding of the neurobiological underpinnings of AUD, especially in the context of early life adversity and its impact on BMI.

## Limitations

There are several unanswered questions that need to be explored in future large cohort studies. One limitation of our study is the use of self-reported questionnaire to measure ELS events. This type of measure is prone to recall bias and may not provide a complete evaluation of ELS. Furthermore, our study did not disentangle the effect of each type of ELS experience, even though research shows that different adverse events may have different effects on brain structure and network connectivity. For example, deprivation and neglect are linked to changes in executive control network regions, such as the dorsolateral prefrontal cortex and parietal cortex, while threat and abuse-related exposures are linked to alterations in regions of the salience network [67]. Additionally, adults who grew up in poverty exhibit reduced activation in the ventrolateral prefrontal cortex and have difficulty regulating emotions [68]. In a recent study alterations in connectivity within the SN was found to mediate the effects of childhood abuse and neglect with problematic alcohol use [69]. Although there are no studies that have directly compared the impact of ELS on connectivity differences with increase in BMI measures in the AUD population, the age at which the stress occurred [70] and the level or duration of stress exposure [71] are crucial factors that should be explored in future studies. Moreover, the correlations observed with ELS and BMI measures with brain connectivity in individuals with AUD (vs. non-AUD) give rise to various conclusions. For instance, it is possible that ELS influences both alcohol abuse and excessive eating. Alternatively, it could be that ELS-driven AUD contributes to overeating, or that ELS-driven overeating increases vulnerability to alcohol overconsumption. The significance of these findings emphasizes the need for longitudinal studies instead of solely relying on cross-sectional research. It is also crucial to conduct longitudinal studies that follow individuals with AUD from an early stage, allowing the observation of potential changes in their brain patterns over time, particularly in relation to any fluctuations in BMI. Another potential limitation is that AUD treatment seekers were administered various medications during their in-patient stay, including anti-depressants, anti-psychotic agents, smoking cessation agents, antihypertensive agents or an attention deficit hyperactivity disorder therapy agent, which may have influenced the functional connectivity results (please refer to Table 1); however, since these were absent from the non-AUD control group, it was not appropriate to include these as covariates. Lastly, our identified connectivity patterns between SN seeds and other brain networks does not align with many other studies on the effects of ELS on SN seed-based connectivity. This may be attributed to the relatively low severity of the stressors reported in our cohort, making direct comparisons with previous results difficult. In our forthcoming study, we aim to investigate potential sex effects that may be influencing the observed correlations and relationships. Furthermore, it is crucial to replicate our findings using large datasets to assess the consistency and reliability of the results, reinforcing the significance and validity of our study’s outcomes.

## Conclusion

To conclude, we identified positive correlations in the connectivity of our SN seeds with clusters associated with emotion, self-regulation, decision-making and impulsivity for salient stimuli in an AUD *vs*. non-AUD population which correlated positively with a history of ELS-related events and BMI measures. Further our results revealed the impact of the interaction of ELS events and BMI elevation on the observed brain connectivity patterns in study participants with AUD (*vs*. non-AUD). These findings underscore the significance of ELS and BMI in modulating the SN seed connectivity in AUD and its role in the neurobiological mechanisms that drive AUD. The results from our study suggest potential directions for future large-scale research on the neural mechanisms for the comorbid occurrence of obesity in individuals with AUD with a history of ELS. This might facilitate the development of targeted interventions for such individuals.

### Supplementary information

Supplemental Figure S1

## Data Availability

The information analyzed in this study from the NIAAA is bound by specific licenses and restrictions. The dataset is under the care and control of the NIAAA Office of the Clinical Director and is securely housed there. Dataset access requests are directed to Melanie Schwandt, melanies@mail.nih.gov. The corresponding authors can provide the findings of this study upon receiving a reasonable request.
