# Supplementary material for: Early life stress and body-mass-index modulate brain connectivity in alcohol use disorder
Source: Transl Psychiatry. 2024 Jan 20;14:43. doi: 10.1038/s41398-024-02756-8 (PMC10799859; doi:10.1038/s41398-024-02756-8)
Supplement: Supplementary file 1 — Supplemental Figure S1 [file 41398_2024_2756_MOESM1_ESM.docx]

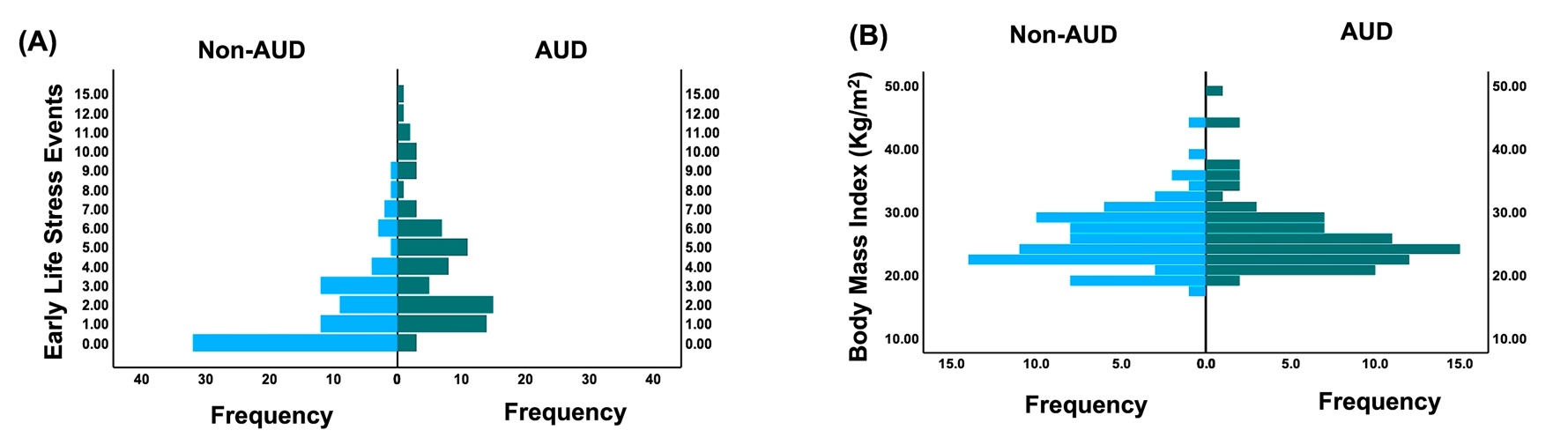


**Supplemental Figure S1. Early life stress (ELS) events and body mass index (BMI) distributions in NIAAA inpatient alcohol use disorder (AUD) vs. non-AUD cohort.** The figures illustrate the distribution of early life stress (ELS) Events (A) and body mass index (BMI) measurements (B) in the NIAAA inpatient AUD treatment-seeking vs. non-AUD cohort who underwent resting-state functional connectivity (rs-fMRI). Frequencies for ELS events were calculated in 1.0 event interval, upto 15 events and for BMI in 10.0 kg/m^2^ intervals, up to 50 kg/m^2^.
